# ProGlide entrapment of the occlusive balloon during repair of an iatrogenic subclavian artery injury

**DOI:** 10.1259/bjrcr.20230015

**Published:** 2023-09-12

**Authors:** Benjamin JR Kemp, Daniel J Kearns, Raman Uberoi

**Affiliations:** 1 Oxford University Hospitals, Oxford, United Kingdom

## Abstract

The insertion of any central venous catheter (CVC) is associated with a risk of damage to neurovascular structures, pneumothorax, cardiac arrhythmias, and infection^1^. Unintentional arterial puncture remains rare, occurring in 6.3–9.4% of attempted internal jugular vein (IJV) catheterisation and 3.1-4.9% of attempted subclavian vein catheterisation^2^.

We present a previously undocumented complication encountered while utilising the Perclose ProGlide device in the case of a 59-year-old male who underwent right subclavian artery closure following the accidental insertion of a 14Fr Vascath into the right subclavian artery. This was performed using two ProGlide devices and one Angio-Seal device. Following deployment of the ProGlide devices, an uninflated balloon passed into the subclavian artery as a precaution, but not used, was removed. One of the ProGlide devices became dislodged having been deployed into the balloon, threatening haemostasis.

## Clinical presentation

A 59-year-old male with a history of ischaemic heart disease was admitted to cardiology with suspected decompensated heart failure exacerbated by community acquired pneumonia. This was further complicated by deranged liver function and clotting, the likely result of ischaemic liver injury. Despite inotropic support and intravenous antibiotics, the patient continued to deteriorate over the subsequent 24 h and a decision was made to admit the patient to ICU. It was decided to place a right-sided internal jugular vein CVC for central venous pressure monitoring and intravenous inotropes. A 14Fr Vascath (BD Bard, New Providence, NJ, USA) was inserted due to the potential requirement for dialysis. No immediate complications were identified.

## Investigations/Imaging findings

**Figure 1.**
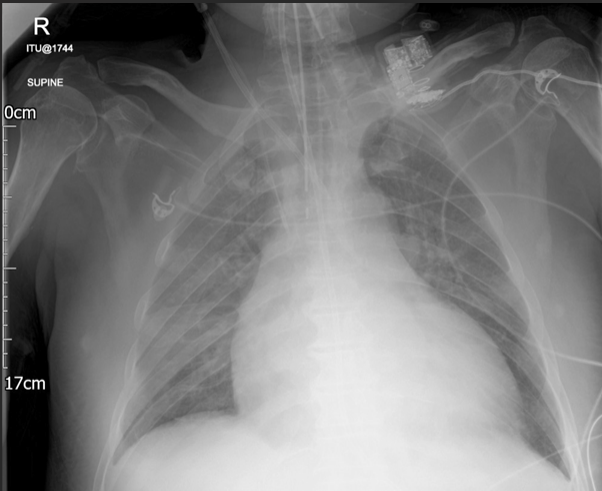
The misplaced large-bore Vascath is projected to the left of the trachea with the tip in the brachiocephalic artery. Note the correctly placed right internal jugular CVC.

Due to a clinical concern of possible arterial insertion, a blood gas was taken from the Vascath, and this was consistent with arterial insertion. The tip of the CVC was found to be projected to the left of the trachea on a chest X-ray performed later the same day, further raising suspicion for accidental puncture of the right common carotid or subclavian artery (Figure 1). A CT angiogram confirmed that the Vascath had traversed the IJV and punctured the right subclavian artery, with the tip located at the origin of the brachiocephalic trunk. There was no suspicion of an intimal flap or haematoma on this examination. The CT angiogram also confirmed a dominant right vertebral artery.

The patient was discussed at the vascular multidiscplinary team meeting (MDT) and a decision was made to perform an endovascular repair of the punctured right subclavian artery. A consensus was also reached regarding the best method of repair, and it was felt that the use of closure devices was preferable to insertion of a covered stent due to the risk of occluding the adjacent dominant vertebral artery. The endovascular repair was delayed allowing some improvement in the patient’s coagulopathy.

## Treatment

**Figure 2.**
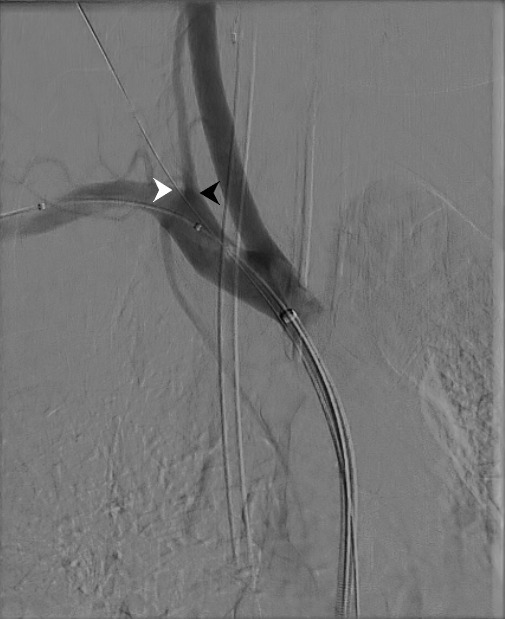
Following removal of the Vascath over a wire the proximity of the puncture site (white arrow) to the origin of the right vertebral artery (black arrow) is clear. Note the uninflated ‘safety-balloon’ within the right subclavian artery.

**Figure 3.**
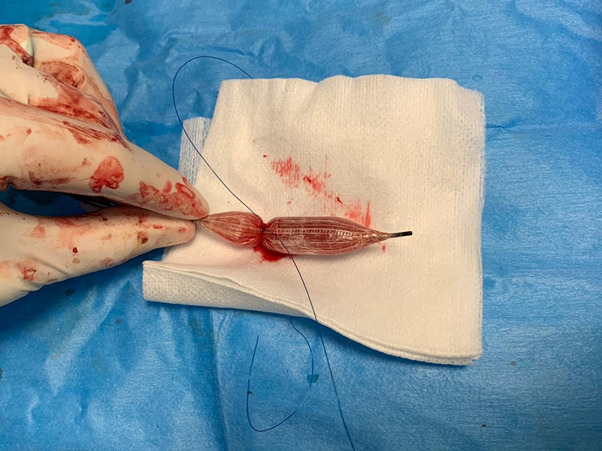
The ProGlide device deployed within the ‘safety-balloon’. This was removed without incident from the femoral artery puncture site.

Access was gained via the left common femoral artery and an 8Fr sheath was inserted to the brachiocephalic artery. An uninflated 14 mm balloon was passed into the right subclavian artery for safety, in case the closure device failed to obtain haemostasis (Figure 2). The distal tip of the Vascath was found to be subintimal and therefore a Glidewire (Terumo, Tokyo, Japan) was passed via a more proximal lumen into the descending thoracic aorta before being exchanged for a 0.35 Amplatz super stiff guidewire (Boston Scientific, Marlborough, MA, USA). The Vascath was removed and two ProGlides (Abbott, Abbott Park, IL, USA) were deployed, not achieving haemostasis and these were augmented with a 6Fr Angio-Seal (Terumo, Tokyo, Japan) and haemostasis was achieved. On removal of the ‘safety-balloon’, one of the ProGlide sutures became dislodged and was found to have deployed into the balloon. The balloon was successfully removed entirely intact via the groin sheath with the ProGlide suture deployed into the uninflated balloon (Figure 3). Subsequent angiography revealed a small focal filling abnormality thought to represent pseudoaneurysm. This was treated with prolonged low-pressure inflation with a new 14 mm balloon.

## Outcome and Follow-up

A CT angiogram performed the following day demonstrated small residual dissection flaps in the brachiocephalic and subclavian arteries and a non-occlusive thrombus in the proximal right axillary artery. The subclavian artery was repaired successfully, unfortunately the patient had suffered cerebral infarctions in the days before removal of the CVC (which we know is true) and eventually died after a long admission under the stroke team.

## Discussion

Numerous endovascular treatment options are described for management of inadvertent arterial puncture following CVC insertion. These include tract embolisation, balloon tamponade, insertion of covered stents and the use of closure devices.^3^ The specific method of closure employed is guided by numerous patient factors including the length of the subcutaneous tract, proximity of the puncture site to branching vessels and the presence of arterial wall dissection or pseudoaneurysm. The use of closure devices is typically considered in the absence of direct branch vessel involvement and dissection.^3,4^ Previously described cases have also employed a ‘safety-balloon’ through a separate puncture site in case of failure of the closure device.^4^

The Perclose Proglide device has a well-documented history of ‘off-label’ use for the closure of accidental subclavian artery puncture sites.^4,5^ There are also reported cases in which collagen-plug closure devices (*e.g.,* Angio-Seal) have been successfully deployed for the management of this complication.^6,7^ Repair of unintentional subclavian puncture using closure devices is often preferred over open surgical options. The primary reason for this is that CVC insertion is often indicated for critically unwell patients whose fitness for surgery is compromised.^3^

The use of vascular closure devices for the repair of inadvertent arterial punctures has both a low rate of immediate and long-term complications, 2 and 3%, respectively.^8^ It is worth noting that most reported repairs follow iatrogenic puncture with catheters ranging from 6 to 9Fr. Our repair, following the insertion of a 14Fr Vascath, therefore warranted the use of two ProGlide devices and augmentation with a Angio-Seal device.

The most commonly utilised approach involves vascular access at another site to perform angiography and for the deployment of a ‘safety-balloon’ should haemostasis not be achieved.^4–7^ This was the technique employed in our case. From our experience, we would recommend having a sheath and guidewire in place but to only advance the balloon if required following the following deployment of the ProGlide sutures. This method allows for prompt balloon tamponade in cases where haemostasis is not achieved without the risk of inadvertent balloon puncture.

## Learning points

- Numerous treatment options are available for the management of iatrogenic arterial injury; vascular MDT should be considered in cases of significant injury or when there is doubt as to the best approach.
- The use of closure devices may be preferred over open repair in unwell or comorbid patients provided there is no branch vessel involvement or significant dissection.
- The use of a ‘safety-balloon’ is prudent to avoid significant bleeding in the event of device failure but this should be kept uninflated and away from the closure site to avoid inadvertent entrapment.
